# Model matchmaking via the Solve-RD Rare Disease Models & Mechanisms Network (RDMM-Europe)

**DOI:** 10.1038/s41684-024-01395-2

**Published:** 2024-06-24

**Authors:** Kornelia Ellwanger, Julie A. Brill, Elke de Boer, Stephanie Efthymiou, Ype Elgersma, Marynelle Icmat, François Lecoquierre, Amanda G. Lobato, Manuela Morleo, Michela Ori, Ashleigh E. Schaffer, Antonio Vitobello, Sara Wells, Binnaz Yalcin, R. Grace Zhai, Marc Sturm, Birte Zurek, Holm Graessner, Eva Bermejo-Sánchez, Teresinha Evangelista, Nicoline Hoogerbrugge, Vincenzo Nigro, Rebecca Schüle, Alain Verloes, Han Brunner, Philippe M. Campeau, Paul Lasko, Olaf Riess

**Affiliations:** 1 Institute of Medical Genetics and Applied Genomics, University of Tübingen, Tübingen, Germany; 2 The Hospital for Sick Children, Toronto, Ontario Canada; 3 Department of Molecular Genetics, University of Toronto, Ontario, Canada; 4 Department of Human Genetics, Radboud University Medical Center, Nijmegen, the Netherlands; 5 Donders Institute for Brain, Cognition and Behaviour, Radboud University Medical Center, Nijmegen, the Netherlands; 6 Department of Neuromuscular Disorders, Queen Square Institute of Neurology, University College London, London, UK; 7 Department of Clinical Genetics, Erasmus Medical Centre, Rotterdam, the Netherlands; 8 Normandie Univ, UNIROUEN, Inserm U1245, CHU Rouen, Department of Genetics, FHU G4 Génomique, Rouen, France; 9 Department of Molecular and Cellular Pharmacology, University of Miami Miller School of Medicine, Miami, FL USA; 10 Graduate Program in Human Genetics and Genomics, University of Miami Miller School of Medicine, Miami, FL USA; 11 Telethon Institute of Genetics and Medicine (TIGEM), Pozzuoli (Na), Italy; 12 Department of Precision Medicine, University of Campania Luigi Vanvitelli, Napoli, Italy; 13 Department of Biology, University of Pisa, Pisa, Italy; 14 Department of Genetics and Genome Sciences, Case Western Reserve University, Cleveland, OH USA; 15 INSERM-Université de Bourgogne UMR1231 GAD «Génétique Des Anomalies du Développement», FHU-TRANSLAD, UFR Des Sciences de Santé, Dijon, France; 16 Dijon University Hospital- UF innovation en diagnostic génomique, Dijon, France; 17 The Mary Lyon Centre at MRC Harwell, Harwell Science Campus, Oxon, UK; 18 Inserm Unit 1231, University of Bourgogne Franche-Comté, Dijon, France; 19 Institute of Rare Diseases Research (IIER), Instituto de Salud Carlos III (ISCIII), Madrid, Spain; 20 Sorbonne Université, Inserm, Institut de Myologie, Centre de Recherche en Myologie, Paris, France; 21 Department of Neurodegeneration, Hertie Institute for Clinical Brain Research (HIH), University of Tübingen, Tübingen, Germany; 22 Department of Genetics, Assistance Publique-Hôpitaux de Paris - Université de Paris, Robert DEBRE University Hospital, Paris, France; 23 Department of Pediatrics, CHU Sainte-Justine and University of Montreal, Montreal, Quebec, Canada; 24 Department of Biology, McGill University, Montreal, Quebec Canada; 25 Present Address: Department of Neurology, University of Chicago, Chicago, IL USA

**Keywords:** Experimental models of disease, Disease genetics, Functional genomics

## Abstract

In biomedical research, particularly for rare diseases (RDs), there is a critical need for model organisms to unravel the mechanistic basis of diseases, perform biomarker studies and develop potential therapeutic interventions. Within Solve-RD, an EU-funded research project with the aim of solving large numbers of previously unsolved RDs, the European Rare Disease Models & Mechanisms Network (RDMM-Europe) has been established.

## Europe contributes to the international RDMM network

RDMM-Europe is a brokerage service to promote fruitful collaborations between clinicians and model organism experts with the aim of filling the gap between RD gene discovery and functional validation of potentially new disease genes and/or novel disease mechanisms (Box 1). For this purpose, RDMM-Europe catalyzes the connection of Solve-RD clinicians and scientists, who have discovered new disease-causing genes, with model organism investigators (MOIs), who are experts for the given genes, the proposed model organism and/or cell culture systems. Solve-RD provides seeding grants for selected validation projects to be conducted by researchers outside the consortium across the world.

RDMM-Europe follows the steps of and makes use of the infrastructure provided by the successful Canadian RDMM Network^1^. Both networks are built on a registry used as the central model-matchmaking platform. The RDMM-Europe Registry (https://rdmm.imgag.de) is a database that allows all interested MOIs to register their genes of interest and the respective model organisms or systems they work with. Registrants thereby express interest in getting connected with scientists and/or clinicians representing patients with a RD and in collaborating in seed-funded validation projects provided by Solve-RD. Registered users have the option to share their data publicly, and these data are accessible to any interested user through the public search interface. The RDMM-Europe Registry is also linked to other international partner network registries such as the Canadian RDMM network (http://www.rare-diseases-catalyst-network.ca/), the Australian Functional Genomics Network (https://www.functionalgenomics.org.au/), the Japanese RDMM network (https://j-rdmm.org/) and the global matchmaking platform ModelMatcher^2^, thereby forming a global network of RDMM networks (https://rdmminternational.org/) and allowing high interconnectivity across the world.

RDMM-Europe has been established by Solve-RD (https://solve-rd.eu/), an EU-funded research project with the primary goal of studying large numbers of unsolved RD for which a molecular cause has not been identified yet^3^. More than 70% of RD cases are predicted to have a genetic cause^4^. However, less than 50% of all patients with RD receive a genetically confirmed diagnosis with the currently applied diagnostic methodologies^5^, leaving more than 15 million European patients with RD without a diagnosis. Consequently, the primary focus of Solve-RD is to explore the diagnostic potential of re-analyzing existing exome and genome data using standardized and novel bioinformatic algorithms and to investigate the use of novel omics technologies and methods such as RNA sequencing, whole genome sequencing (WGS), long-read sequencing and metabolomics for RD research. In Solve-RD and beyond, this approach has successfully increased the diagnostic sensitivity of whole exome sequencing (WES)/WGS analysis (unpublished data S.L.), but also facilitated the discovery of potential novel disease genes^6,7^. Newly identified disease-causing genetic variants are extremely rare, stimulating worldwide efforts to identify further families affected by these variants and to decipher the role of a novel gene in the disease via the Matchmaker Exchange platform, a federated network of RD databases allowing novel disease–gene relationship discoveries^8,9^. Even with these collaborative efforts, identifying additional families remains difficult. Hence, programs supporting functional analyses are urgently needed to provide additional evidence that a candidate gene is causative for the respective disease. Here, RDMM-Europe has a pivotal role by catalyzing new collaborative projects with RD experts and supporting research with model organisms and systems for functional validation of genes of interest.

Box 1 Key facts about the RDMM-Europe brokerage service **Objective:**

Recruitment of scientific expertise that is not present within the Solve-RD consortium.

**Focus:**

Molecular and functional validation of newly identified genes or novel disease mechanisms.

**Funding:**

Seeding grants (20,000 Euro per project/gene) for collaborative projects provided by Solve-RD.

## The RDMM-Europe model matchmaking pipeline

**Fig. 1:**
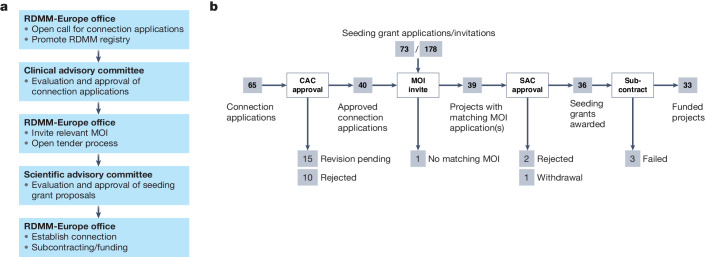
The RDMM-Europe model-matchmaking pipeline. **a**) Steps for the application and selection process managed by the RDMM-Europe Office and the two selection committees. **b**) From 2019 to 2022 the RDMM-Europe Office opened 10 calls for connection applications and received a total of 65 proposals. All connection applications were reviewed by the Clinical Advisory Committee (CAC) which either approved (n=40), rejected (n=10) or requested to revise (n=15) the proposals. The RDMM-Europe Office opened a call for tender for 39 of the approved connection applications and invited in total 178 model organism investigators (MOI), of which 73 submitted a seeding grant application. Those seeding grant applications covered 39 of the genes that were approved for functional validation by the RDMM program. The Scientific Advisory Committee (SAC) evaluated all 73 seeding grant applications and approved 36 proposals for funding. For one approved candidate gene, the clinical group withdrew their original validation request. Two other validation requests were rejected by the SAC. For three projects, subcontracting subsequently failed, resulting in a total of 33 funded projects.

To promote the connection between RD researchers and clinicians, a two-committee process was implemented (Fig. 1a). Connection applications for novel RD candidate genes are submitted by clinicians or scientists from one out of the four associated European Reference Networks (ERN) for Rare Diseases that form the clinical core of Solve-RD^3^. Connection applications are evaluated and approved by the Clinical Advisory Committee (CAC) in a process that starts with peer review and is followed by general discussion by the whole CAC. The CAC is composed of clinical genomics experts from the ERNs and the undiagnosed rare disease programs involved in Solve-RD (https://solve-rd.eu/the-group/consortium/). The committee evaluates proposals based on defined criteria (http://solve-rd.eu/wp-content/uploads/2020/09/Connection-Application-Form_v6.docx) and decides which candidate genes should be considered for connection via the RDMM program. Upon approval of a candidate gene, the RDMM-Europe management office opens a call for tender to identify the best match among MOIs for functional gene validation, and these scientists are invited to submit a short seeding grant application. For model matchmaking and to identify the best MOI, existing RDMM registries are used; the process includes looking into international partner networks and other model matchmaking initiatives that Solve-RD is connected with^10^, complemented by classical search options, e.g., via publication databases. The Scientific Advisory Committee (SAC) evaluates the seeding grant applications received and approves projects for funding. In general, only one seeding grant is promoted per disease gene.

Overall, since December 2019, ten calls for connection applications have been opened at three-month intervals. Proposals were selected according to the above-mentioned process (Fig. 1). In total, 65 connection applications were submitted, and 40 of them were positively evaluated and approved by the CAC (Fig. 1b). The RDMM-Europe Office opened a tender process for each of the approved genes. To be in line with German procurement law (the coordinating office of RDMM-Europe is located in Germany) and to increase the chance of finding the best possible match, at least three independent MOIs were invited to propose functional validation projects for each gene. In total, 178 researchers were invited to submit a seeding grant application, and 73 of them subsequently submitted proposals. These applications covered 39 out of the 40 approved genes. The SAC evaluated all received seeding grant proposals and approved 36 projects, which best addressed the aim of validation requested by the clinical research group and offered the highest likelihood of providing new functional evidence for the gene variants in the context of the given disease. Upon approval of a project by the SAC, the RDMM-Europe Office initiated the conclusion of a subcontract between the Solve-RD lead beneficiary and the institution of the scientific modeling group, which in principle can be located anywhere in the world. Funding approval was concluded following three rounds of scientific review or selection (CAC approval, MOI invite, SAC approval), each taking 3–4 weeks of decision time, averaging to a total time from the initial application to the funding commitment of less than three months. Of note, three projects failed subcontracting, ending in a total of 33 collaborative modeling projects funded by the Solve-RD budget.

## New international connections to advance RD research

In this section, we showcase the main objectives of the funded projects, highlight examples of validation projects in different models and provide links to recent publications related to some of these projects^11–14^ (Table 1 and Supplementary Information detailing six specific use cases). All projects aim to functionally validate a gene with respect to the given pathology (Table 1). Some projects are also expected to provide insights into the mechanisms of pathogenicity (mechanism) and/or therapeutic avenues (treatment).

**Table 1:** Details of international projects established and supported by RDMM-Europe

Disease	ERN	Location of subcontractor	Scope	Model organism(s)	Reference
Developmental disorder	ITHACA	Rotterdam, the Netherlands	Gene validation; mechanism	Mouse embryo	Supplementary Fig. S1 and^14^
Malformative syndrome without intellectual disability representing distinct entity from Pitt-Hopkins syndrome	ITHACA	Pisa, Italy	Gene validation; mechanism; treatment	*Xenopus* & zebrafish	Supplementary Fig. S2
Cortical dysplasia, complex, with other brain malformations	ITHACA	Dijon, France	Gene validation; mechanism	Mouse	Supplementary Fig. S3
Demyelinating neuropathy with central involvement	RND	Oxon, UK	Gene validation; mechanism	Mouse	Supplementary Fig. S4
Microcephaly, developmental delay and facial dysmorphisms	ITHACA	Toronto, Canada	Gene validation; mechanism	*Drosophila*	Supplementary Fig. S5
Autosomal recessive hereditary spastic paraplegia (HSP)	RND	Miami, US	Gene validation; mechanism; treatment	*Drosophila*	Supplementary Fig. S6
Acute middle age onset respiratory insufficiency with selective muscle involvement	Euro-NMD	Washington, D.C., US	Gene validation	Mouse	^11^
Polymalformative syndrome	ITHACA	Toronto, Canada	Gene validation; mechanism	*Drosophila*	-
Neurodevelopmental disorder	ITHACA	Oklahoma City, US	Gene validation	Zebrafish	-
Distal myopathy with some proximal involvement	Euro-NMD	Helsinki, Finland	Gene validation	Zebrafish	-
Neurodevelopmental disorders	ITHACA	Ghent, Belgium	Gene validation; mechanism	*Drosophila*	^13^
Hereditary spastic paraplegia, subtype SPG32	RND	Paris, France	Gene validation	Zebrafish	-
Intellectual disability and autism spectrum disorder	ITHACA	Lyon, France	Gene validation	*Caenorhabditis elegans*	-
Neurodevelopmental disorder	ITHACA	Milano, Italy	Gene validation	iPSC	-
Serrated Polyposis Syndrome (SPS)	GENTURIS	Cleveland, US	Gene validation	Cell–cell adhesion assay; mouse	-
Myopathy with severe (lethal) cardiac involvement	Euro-NMD	Sydney, Australia	Gene validation	Patient-derived cells	-
Intellectual disability	ITHACA	Oxon, UK	Gene validation	Mouse	-
Cornelia de Lange-related disorder	ITHACA	Doha, Katar	Gene validation	Zebrafish	-
Epileptic encephalopathy	Euro-NMD	Osaka, Japan	Gene validation; mechanism	Mouse	-
Global developmental delay, cardiac involvement and facial dysmorphism	ITHACA	Prague, Czech Republic	Gene validation	Mouse & zebrafish	-
Severe developmental delay, seizures and facial dysmorphisms	ITHACA	Prague, Czech Republic	Gene validation	Mouse & zebrafish	-
Developmental disorder	ITHACA	Munich, Germany	Gene validation	Mouse	-
Neurodevelopmental disorder (NDD)	GENTURIS	London, UK	Gene validation; mechanism; treatment	Zebrafish	-
Microcephaly, global developmental delay and craniofacial abnormalities	ITHACA	Paris, France	Gene validation; mechanism	Zebrafish	-
Complex dystonia-parkinsonism-ataxia phenotype	Euro-NMD	Oklahoma City, US	Gene validation	Zebrafish	-
A new genetic form of Hereditary Spastic Paraplegia (HSP)	Euro-NMD	Rotterdam, the Netherlands	Gene validation; treatment	Zebrafish	^12^
Polymalformative syndrome	ITHACA	Ghent, Belgium	Gene validation; mechanism	*Drosophila*	-
Medullary thyroid cancer and breast cancer	GENTURIS	Budapest, Hungary	Gene validation	Zebrafish	-
Autosomal-dominant cerebellar ataxia	RND	Massachusetts, US	Gene validation	Recombinant protein assay	-
Epileptic encephalopathy with severe brain degeneration	Euro-NMD	Geneva, Switzerland	Gene validation; treatment	*Drosophila*	-
Non-syndromic sensorineural hearing impairment	Euro-NMD	Göttingen, Germany	Gene validation	Mouse	-
Developmental and epileptic encephalopathy	Euro-NMD	Manitoba, Canada	Gene validation; mechanism; treatment	*Drosophila*	-
A distinct syndromic neurodevelopmental disorder	Euro-NMD	Ghent, Belgium	Gene validation	*Xenopus* & zebrafish	-

For each project, the table provides disease description, the European Reference Network for rare diseases (ERN) of the clinical partner that proposed a gene for validation, the geographic location of the seeding grant recipients (subcontractor), the main scope of the project, the model organism(s) and link to related publications. Further details about the first 6 projects are given in the Supplementary information. All other projects are listed in the order of the formal start dates defined by the conclusion of the subcontracts. RD, rare disease; RND, rare neurological diseases; ERN ITHACA, ERN for rare malformation syndromes, intellectual and other neurodevelopmental disorders; EURO-NMD, ERN for rare neuromuscular diseases; ERN GENTURIS, ERN for genetic tumor risk syndromes; iPSC: induced pluripotent stem cell.

Until now, seeding grant funding has been provided for 33 projects, and the initiative has connected Solve-RD clinicians and scientists to MOIs located in ten different European countries (Belgium (n = 3), Czech Republic (n = 2), Germany (n = 2), Finland (n = 1), France (n = 4), Italy (n = 2), the Netherlands (n = 2), Hungary (n = 1), Switzerland (n = 1), and the UK (n = 3)), as well as in the US (n = 6), Canada (n = 3), Qatar (n = 1), Japan (n = 1), and Australia (n = 1). In terms of model organisms and systems, zebrafish proved to be the most popular model within the projects, followed by mice and *Drosophila*. Four projects applied combined approaches using more than one model system (Table 1).

Once all projects are completed, we will evaluate the different modeling approaches to accelerate new disease–gene associations, better understand the pathophysiological mechanisms of ultra-rare and complex diseases and identify and develop potential therapeutic approaches for the benefit of patients living with RD.

## Conclusions and future steps

RDMM-Europe, with its international integration into the worldwide RDMM network, is an important actor for achieving the aims of Solve-RD, which are to find novel disease genes and define disease mechanisms for ultra-RDs. Major advantages of RDMM are: a) low (no) hurdles for receiving seeding grants; b) concise applications; c) short decision times; d) standardized evaluation protocols and a transparent decision process; and e) bringing world experts together.

By experimentally confirming new disease genes, RDMM serves the international goals of improving knowledge on RD while decreasing the time until a diagnosis is made for patients with RD. Although the number of projects has been restricted by the end of the Solve-RD funding period, the general demand for connection might be considerable because almost all of the newly identified causes of RD are ultra-rare^15^. In Solve-RD, we initially could only serve major clinical indications as defined by the core ERNs (ERN-ITHACA, ERN-RND, ERN-Euro NMD, ERN-GENTURIS), but over time Solve-RD integrated many more ERNs and Undiagnosed Diseases Networks (UDN) as partners. Ultimately, 24 ERNs have been established in Europe, and numerous clinicians and scientists have successfully identified specific genes involved in ultra-RDs. Thus, the need for models to functionally validate their findings is steadily increasing.

Based on genome data and (long-read) WGS analysis, scientists are expecting a trend toward the identification of novel disease mechanisms (such as mutations within non-coding regions, enhancers and topologically associating domains (TADs)), which may be more difficult to study compared to open reading frame alterations; and also, a strong movement into epigenomics. Therefore, it is anticipated that hundreds or even thousands of novel disease genes and/or disease mechanisms will be discovered over the next decade. Given that most of these diseases are expected to be ultra-rare, establishing patient cohorts of suitable size with the same genetic cause might be difficult, further increasing the need for modeling in cell systems or animals. In addition, disease modification is an increasingly important issue in RD discovery. Numerous diseases, such as dystonia (reduced penetrance) or neurofibromatosis (reduced expressivity), might barely manifest in some mutation carriers while leading to severe phenotypes in other patients, even in the same family. Given that no obvious environmental factors have been found to explain these discrepancies, one may conclude that genetic modifiers are the main cause for this phenotypic diversity. Identification and modeling of these variants in different systems/organisms might be critical for future RD research, including for developing new treatment concepts. By the end of the grant, it will be critical to retrospectively evaluate what types of models were the most successful, for instance, in yielding publications; and, probably even more important, what seed fundings were not successful and what can be learned from these failures.

It will also be important to discuss whether the existing call for models and model groups will meet the requirements of projects for the next few years. Indeed, it might be more efficient in terms of time and money to select constant partners for model organisms or specific disease mechanisms. And finally, in the interest of the patients waiting for a diagnosis, we also have the responsibility to functionally investigate variants of clinically unknown significance (VUS) of known disease genes to improve diagnostic sensitivity and specificity. Efficient concepts in this direction must be developed across the world.

Ultimately, the need to validate newly identified variants and genes is driven by the expectations of patients with RD awaiting a diagnosis. Most of these patients are being seen by the clinical expertise centers linked to ERNs, or Undiagnosed Disease Programs, which are involved in Solve-RD. Solve-RD showcases that European collaboration can help address RD research needs. In this sense and more concretely, RDMM-Europe can also be considered a collaboration broker — one, however, that goes beyond European borders.

### Supplementary information

Supplementary Information Supplementary Use Cases and Supplementary Figs. 1–6.
