# Supplementary material for: Model matchmaking via the Solve-RD Rare Disease Models & Mechanisms Network (RDMM-Europe)
Source: Lab Anim (NY). 2024 Jun 24;53(7):161–5. doi: 10.1038/s41684-024-01395-2 (PMC11216991; doi:10.1038/s41684-024-01395-2)
Supplement: Supplementary file 1 — Supplementary Use Cases and Supplementary Figs. 1–6. [file 41684_2024_1395_MOESM1_ESM.pdf]

lab animal

Supplementary information

https://doi.org/10.1038/s41684-024-01395-2

Model matchmaking via the Solve-RD Rare Disease Models & Mechanisms Network (RDMM-Europe)

In the format provided by the authors and unedited

Supplementary Information

Use cases and graphical abstracts

Use case 1: Studying neurodevelopmental disorder

International data sharing and collaborative approaches of Solve-RD and ERN-ITHACA led to the identification of 4 individuals from 4 families with neurodevelopmental disorders (NDD) of significant clinical overlap that all share the exact same *de-novo* missense variant in the *FEM1B* gene, not associated to a human disease to date[1]. *FEM1B* encodes the recognition subunit of an E3 ubiquitin ligase involved in multiple processes, including protein homeostasis, and has also recently been identified as a major player in cellular redox sensing and signaling. We hypothesized that this variant led to a new rare developmental disease, and undertook functional validation of this specific missense variation. We used a combined *in vivo* and *in vitro* approach based on the use of patient samples, a cellular model and a mouse model (Figure S1). The patient samples were used to assess the redox status of the cells, while the mouse model was used to assess the impact of prenatal brain overexpression of the mutated form, compared to the wild-type form, on neuronal migration and neuronal arborization[2].

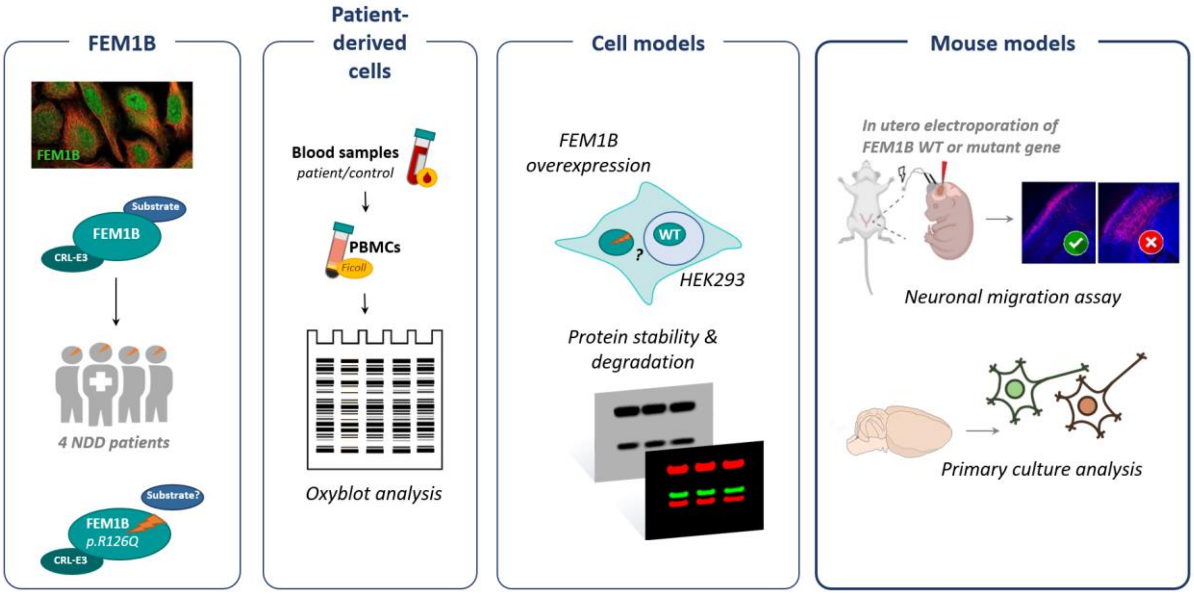

Figure S1: ***In vivo*** **validation of** ***FEM1B*** **associated neurodevelopmental disorder (NDD).** An identical *de novo* missense variant was identified in 4 unrelated NDD patients, raising the hypothesis of a novel disorder mediated by a non-haploinsufficiency mechanism. Functional validation of the effect of this specific missense variant consisted in analysis in patients' cells, cellular overexpression in cell lines and primary mouse neurons, and *in vivo* neuronal migration assays[2].

Use case 2: Studying malformation syndromes

Most projects supported by RDMM-Europe make use of non-mammalian animal models and, among those, zebrafish has proven most popular (Table 1 in main article). We present an example of a combined approach using *Xenopus* and zebrafish models to study *de novo* variants in a gene with a suspected gain-of-function mechanism responsible for a new disease manifesting with congenital malformation syndrome (Figure S2). The molecular machinery controlling craniofacial development is conserved between *Xenopus* and mammals, strengthening the translatability of the results[3]. The model allows targeting wild type or mutated messenger RNA specifically to neural crest cells, which will give rise to craniofacial skeleton, while preserving all the other tissues[3]. Integration of results from gain-of-function experiments in *Xenopus*, and zebrafish with sophisticated -omics analyses in patient-derived samples will help to define the mechanistic role of the gene in development and disease.

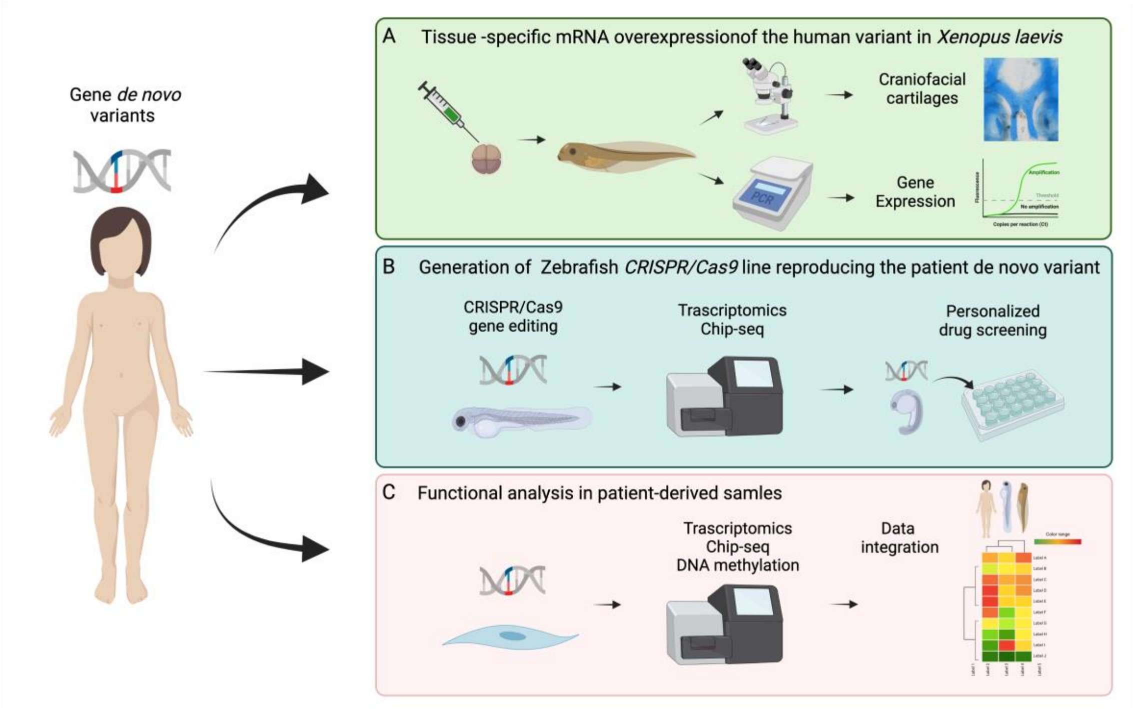

Figure S2: *In vivo* modeling of new gene variants using non-mammalian vertebrates. *Xenopus laevis* and *Danio rerio* (zebrafish) are the gold standard for rapid *in vivo* gene function analysis in vertebrates. A) *Xenopus* embryos can be used to overexpress the mRNA of the wild type or a mutated form of a human gene in a tissue-specific manner. B) Zebrafish can be used for rapid generation of CRISPR/cas9 lines by gene editing, reproducing, when possible, the mutation observed in human patients. These models can be used to unveil new molecular mechanisms associated with the pathology and/or to perform drug screening analyses. C) Combining molecular diagnosis, and omics approaches on patient-derived samples and induced Pluripotent Stem cells (iPSCs) together with gene functional analysis and validation *in vivo*, can contribute to unveil conserved molecular pathway altered in the pathology and new druggable targets (Figure created with BioRender.com).

Use case 3: Novel disease mechanisms

CTNNA2 encodes αN-catenin, a protein involved in connecting the cadherin-catenin complex to cytoskeletal filamentous actin at adherens junctions in the (developing) brain[4, 5]. Homozygous protein truncating variants in *CTNNA2* cause a recessive neuronal migration disorder (MIM #618174) (Figure S3), mechanistically explained by a reduced repression of ARP2/3-initiated actin polymerization, a process normally mediated by the actin binding domain (ABD) of αN-catenin[6]. In Solve-RD, however, heterozygous *de novo* missense variants in *CTNNA2* were uncovered in patients with intellectual disability or neurodevelopmental delay, in absence a second pathogenic allele, hinting towards a dominant inheritance. Interestingly, a subset of patients with *CTNNA2* missense variants affecting the ABD presented with a complex cortical dysplasia phenotype, reminiscent of the recessive disorder associated with *CTNNA2*. The effects of the variants on actin polymerization kinetics are being characterized using recombinant protein assays. Additionally, two knock-in mouse lines have been generated, each carrying one of the *de novo* missense variants, for neuroanatomical and neurobehavioral studies.

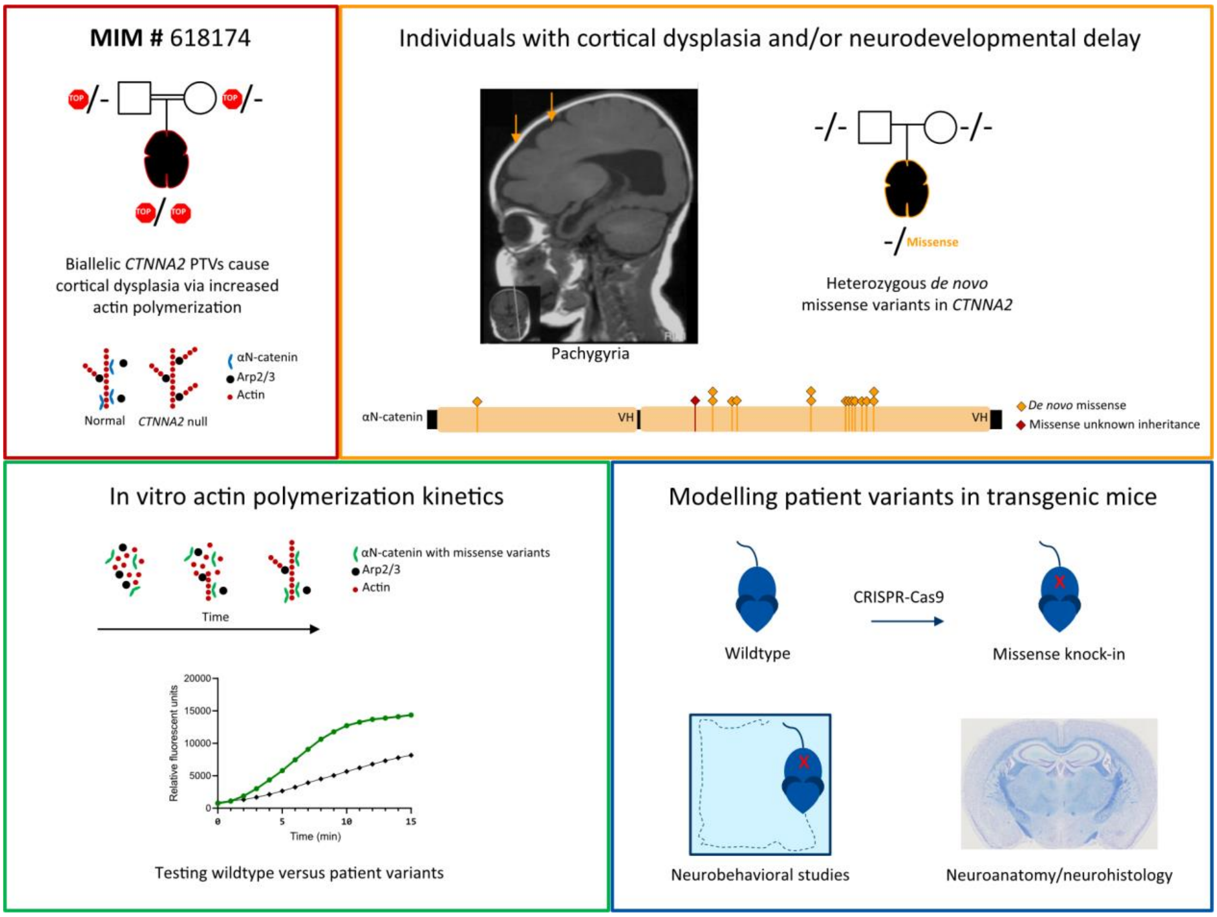

Figure S3: Project parts and experimental design for the *CTNNA2 de novo* missense validation approach. Biallelic protein truncating variants (PTVs) in *CTNNA2* are associated with an autosomal recessive neuronal migration phenotype (MIM #618174; red upper left

panel). Via international collaborations, patients with heterozygous (*de novo*) missense variants in *CTNNA2* and neurodevelopmental phenotypes are identified (orange upper right panel). The observed variants are included in *in vitro* actin polymerization experiments, aiming to elucidate the molecular mechanism underlying the phenotypes (green lower left panel, previously described in[6]). In addition, two knock-in mouse lines are generated with each of the observed missense variants for neurobehavioral and neuroanatomical characterization (blue lower right panel).

Use case 4: Reproduction and rescue of RD phenotypes in animal models

Solve-RD partners identified a novel autosomal-recessive demyelinating neuropathy and NDD in six families exhibiting a spectrum of central (intellectual disability, developmental delay, motor impairment, speech difficulties) and peripheral (early onset demyelinating neuropathy) neurological involvement (Figure S4). Exome and genome sequencing identified one frameshift and five different homozygous non-synonymous variants in *NFASC*. *In vitro* expression studies using immunostaining-based techniques identified absent expression of the Nfasc155 isoform as a consequence of the frameshift variant and a significant reduction of expression was also observed in association with two non-synonymous variants affecting the fibronectin type III domain. Similarly, cell aggregation studies revealed a severely impaired Nfasc155-CNTN1/CASPR1 complex interaction as a result of the identified variants. Additional immunofluorescence staining of myelinated fibres from two affected individuals showed a severe loss of myelinated fibres and abnormalities in paranodal junction morphology (Figure S4). Ongoing *in vivo* functional studies in the mouse aim at the development and phenotypic characterization of a knock-in model of the p.P694T variant.

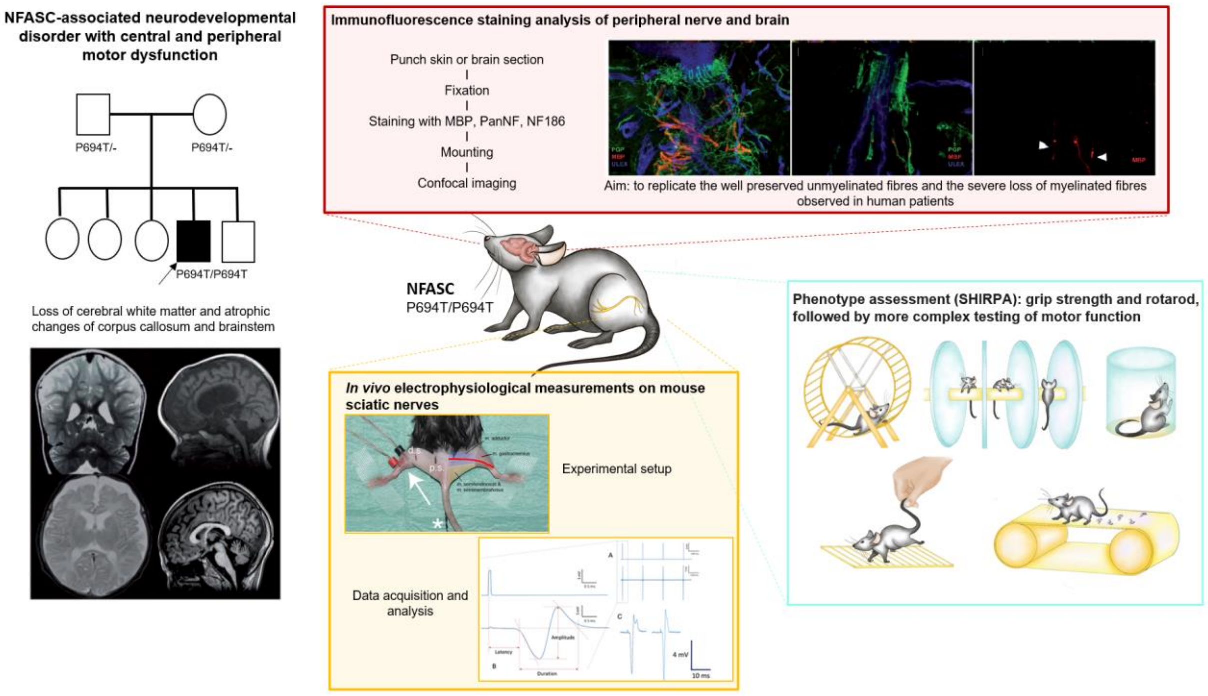

Figure S4: *In vivo* functional investigation of the *NFASC*-associated neuro-developmental disorder with central and peripheral motor dysfunction in a mouse model. A knock-in homozygous p.P694T model is being generated. We plan to use a standard battery of tests, including phenotype assessment (SHIRPA), grip strength and rotarod, followed by more complex testing of motor function using EMG and isometric muscle tension recordings, which can provide compound muscle action potential (CMAP) and motor unit number estimation (MUNE) data, two very important measurements when evaluating nerve conduction in humans and mice. Additionally, immunofluorescence staining of myelinated fibres from mouse-derived nerves will be used to check for abnormalities in the paranodal junction morphology.

Use case 5: Drosophila as a model studying rare diseases

There are a growing number of reports emphasizing the benefit of functional modeling of RD in *Drosophila*[7], and with seven out of 33 validation projects, *Drosophila* is also well represented among the modeling approaches supported by Solve-RD (Table 1 in main article).

As one example, a novel autosomal-dominant syndrome was identified, which is characterized by microcephaly, developmental delay, facial dysmorphisms and visual impairment in seven families carrying heterozygous *de novo* variants in a gene encoding a kinase localized in the nucleoplasm, cytosol and centrosome. Three of the alleles are recurrent variants. Functional studies in *Drosophila melanogaster* are ongoing to provide the evidence needed to support causality and uncover the cellular basis of the disease (Figure S5). Preliminary results demonstrate that high-level expression of both the wild-type and mutated kinase ortholog causes lethality in fly embryos, and that high-level expression of the wild-type kinase in primary spermatocytes causes male sterility. In contrast, low-level expression of the wild-type gene in spermatocytes is compatible with male fertility. For future experiments, low-level promoters will be used for the mutated kinase expression in male germ cells and neuroblasts, and a broader low-level promoter for *Drosophila* S2 cells and whole flies. Moreover, these experiments will determine whether the dominant effects of these mutations require kinase activity.

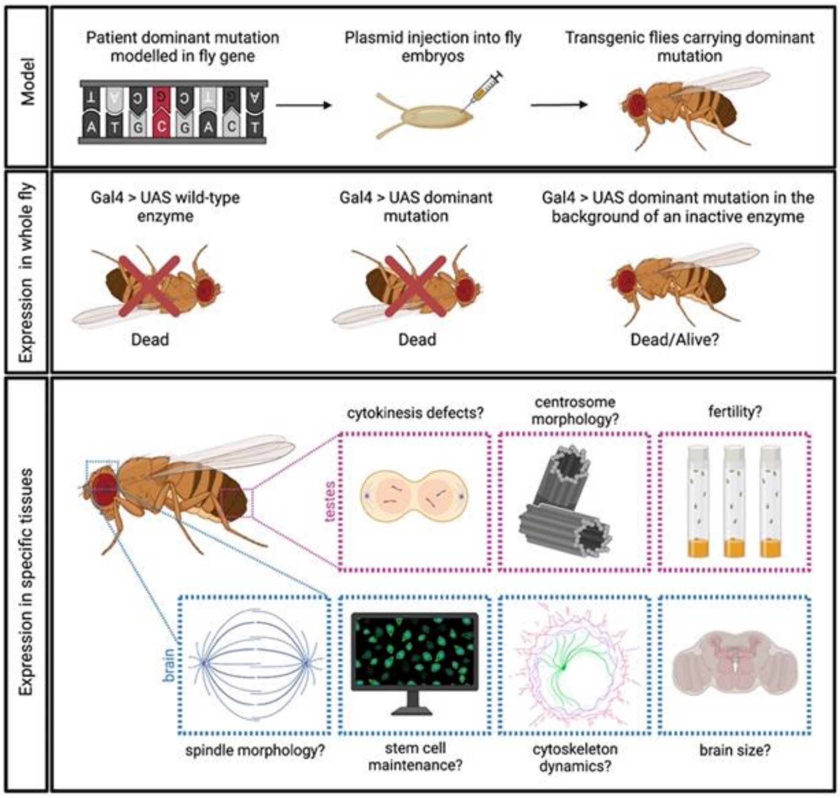

Figure S5: ***Drosophila melanogaster*** **as a model to study dominant disease-causing mutations in humans.** Model (top): Mutations analogous to disease-causing variants found in humans were introduced into the *Drosophila* kinase ortholog to generate transgenic flies carrying the dominant mutations. Expression in whole fly (middle): Preliminary results show that high-level expression of both the wild-type and the mutated kinase results in lethality. Dominant mutations will be introduced into a kinase-dead background to determine if lethality is kinase-dependent. Expression in specific tissues (bottom): To elucidate the nature of the mutations, the mutated kinase will be expressed in different tissues using low-level promoters. Phenotypes in the testis will be characterized by investigating cytokinesis, centrosome morphology and fertility. Meanwhile, spindle morphology, stem cell maintenance, cytoskeleton dynamics and brain size will be examined in flies expressing dominant mutations in the brain (Figure created with BioRender.com).

Use case 6: Modeling towards RD treatments

Besides assessing genotype-phenotype associations, characterization of novel disease-gene associations, and functional disease mechanisms, models can also be used to screen for potential therapeutics by applying drug candidates and exploring if abnormal phenotypes can be rescued[8].

Among a cohort of ~300 WES and WGS datasets from patients with hereditary spastic paraplegia collectively analyzed in Solve-RD, a shared, apparently homozygous, rare missense variant was present in two unrelated index cases. Both index cases are affected by sporadic early onset slowly progressive spastic paraplegia, accompanied by mild cognitive deficits and peripheral neuropathy. Cranial and spinal MRI were unremarkable in both patients,

except mild atrophy of the thoracic spinal cord. The variant co-segregates with the disease in available family members. Searching for additional families with highly similar phenotypic pattern and bi-allelic variants in the same gene via the RD-Connect[9] and the GENESIS[10] platforms revealed three additional matches. In this example, we modeled human diseases in *Drosophila* by generating transgenic flies carrying human cDNA of either wildtype or patient variant (Figure S6). Human transgenes can be expressed in the nervous system in a cell-type specific manner to tease out precisely the underlying disease pathology. By determining the nature of the variant and confirming causality, we can thus identify targets for intervention and treatment. The *Drosophila* model is particularly suitable for identifying such lead compounds for nervous system effects due to its primitive blood-brain barrier that allows drug administration through feeding[11]. Such advantage increases the throughput of compound screening and the potential success rate of identification of efficacious compounds. Subsequent medicinal chemistry approaches can be applied to adjust the chemical properties of the lead compounds and enhance their penetrance through the blood-brain barrier in humans.

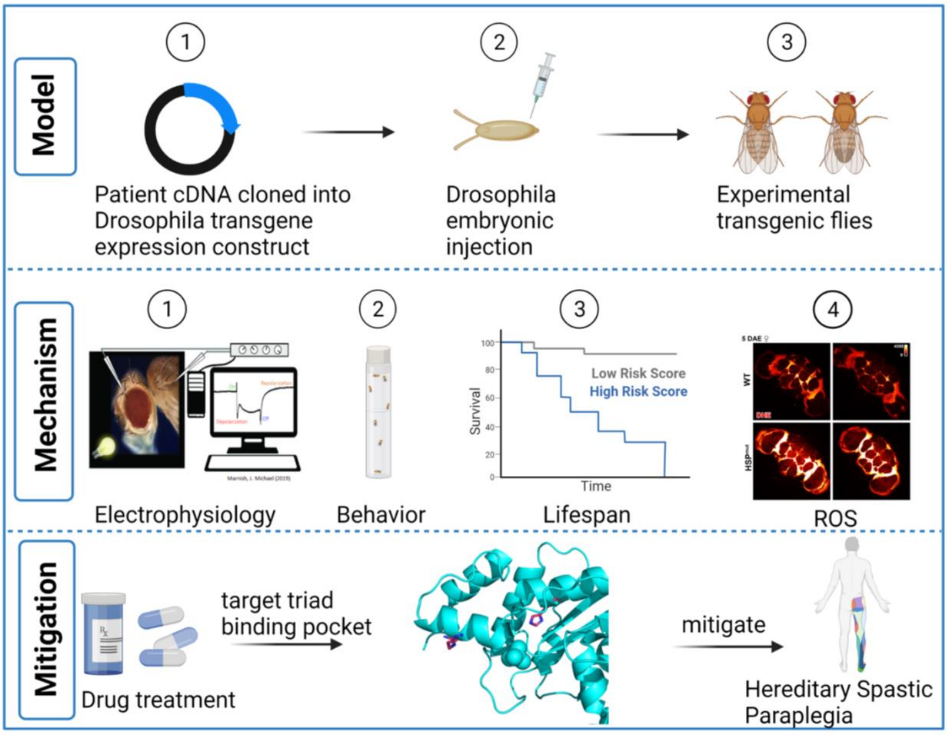

Figure S6: *Drosophila* model addressing disease mechanism and possible therapeutic interventions for Hereditary Spastic Paraplegia (HSP) caused by a novel gene variant. Using *Drosophila* as a model organism to establish the causality for the novel variant identified in HSP (Model), characterize the pathophysiological mechanisms (Mechanism), and identify potential therapeutic targets and drug candidates (Mitigation). Transgenic flies expressing human wildtype or mutant proteins are generated in the loss of endogenous *Drosophila* ortholog background to mimic the human gene expression condition. Pathophysiological changes, behavior, lifespan, and cellular phenotypes of mutant protein expression are compared to the wildtype protein expression to determine the deleterious effect of the mutation

and uncover the underlying mechanism of pathology. Molecular and biochemical analyses on the protein structure containing the novel variant are incorporated to identify therapeutic targets and allow screening of drug candidates for disease mitigation.

References

1. Lecoquierre, F. et al. Variant recurrence in neurodevelopmental disorders: the use of publicly available genomic data identifies clinically relevant pathogenic missense variants. *Genet Med* **21**, 2504-2511 (2019).
2. Lecoquierre, F. et al. A recurrent missense variant in the E3 ubiquitin ligase substrate recognition subunit FEM1B causes a rare syndromic neurodevelopmental disorder. *Genet Med* **26**, 101119 (2024).
3. Corsinovi, D., Giannetti, K., Cericola, A., Naef, V. & Ori, M. PDGF-B: The missing piece in the mosaic of PDGF family role in craniofacial development. *Dev Dyn* **248**, 603-612 (2019).
4. Ishiyama, N. et al. Force-dependent allostery of the α-catenin actin-binding domain controls adherens junction dynamics and functions. *Nat Commun* **9**, 5121 (2018).
5. Borghi, N. et al. E-cadherin is under constitutive actomyosin-generated tension that is increased at cell-cell contacts upon externally applied stretch. *Proc Natl Acad Sci U S A* **109**, 12568-12573 (2012).
6. Schaffer, A.E. et al. Biallelic loss of human CTNNA2, encoding αN-catenin, leads to ARP2/3 complex overactivity and disordered cortical neuronal migration. *Nat Genet* **50**, 1093-1101 (2018).
7. Oriel, C. & Lasko, P. Recent Developments in Using Drosophila as a Model for Human Genetic Disease. *Int J Mol Sci* **19** (2018).
8. Deng, R. et al. AMFR dysfunction causes autosomal recessive spastic paraplegia in human that is amenable to statin treatment in a preclinical model. *Acta Neuropathol* **146**, 353-368 (2023).
9. Laurie, S. et al. The RD-Connect Genome-Phenome Analysis Platform: Accelerating diagnosis, research, and gene discovery for rare diseases. *Hum Mutat* **43**, 717-733 (2022).
10. Gonzalez, M. et al. Innovative genomic collaboration using the GENESIS (GEM.app) platform. *Hum Mutat* **36**, 950-956 (2015).
11. Limmer, S., Weiler, A., Volkenhoff, A., Babatz, F. & Klämbt, C. The Drosophila blood-brain barrier: development and function of a glial endothelium. *Front Neurosci* **8**, 365 (2014).
